# Quality of Life, Pain, and Sexual Desire in the Elderly Over 65 Years of Age

**DOI:** 10.1002/brb3.70655

**Published:** 2025-07-07

**Authors:** Patricia Rocamora‐Pérez, Azahara de la Poza‐Rodríguez, María Jesús Benzo‐Iglesias, José Manuel Aguilar‐Parra, Rubén Trigueros‐Ramos, Remedios López‐Liria, María José Morales‐Gázquez

**Affiliations:** ^1^ Health Research Centre, Department of Nursing, Physiotherapy and Medicine University of Almería Almería Spain; ^2^ Torrecárdenas University Hospital Almería Spain; ^3^ Department of Psychology University of Almería Almería Spain; ^4^ Department of Nursing University of Las Palmas de Gran Canaria (ULPGC) Las Palmas de Gran Canaria Spain

**Keywords:** aged, pain, quality of life, sexual desire, structural equation modeling

## Abstract

**Purpose:**

Sexuality is recognized as a fundamental dimension of human well‐being; however, gaps remain in understanding how changes associated with aging, such as chronic pain, affect sexual desire and quality of life. This study aimed to explore the relationships among quality of life, pain, and sexual desire among people over 65 years of age.

**Method:**

A cross‐sectional design was employed with 400 institutionalized and non‐institutionalized elderly people. Participants completed the Sexual Desire Inventory, Spanish Pain Questionnaire, and WHOQOL‐BREF quality of life scale. Data were analyzed using descriptive statistics, Pearson correlations, and a structural equation model.

**Finding:**

The results revealed a model indicating significant positive correlations between sexual desire and quality of life, suggesting that higher quality of life is associated with greater sexual desire, and vice versa. Additionally, sexual desire correlated negatively with pain, suggesting that greater sexual desire is associated with lower pain levels. Negative correlations were also observed between quality of life—particularly in the physical health domain—and pain. The model fit indices were adequate: *χ* ^2^ (1259, *N* = 400) = 3117.61, *χ* ^2^/df = 2.48, *p* < 0.001, Incremental Fit Index (IFI) = 0.96, Tucker Lewis Index (TLI) = 0.96, Comparative Fit Index (CFI) = 0.96, root mean square error of approximation (RMSEA) = 0.058 (90% confidence interval [CI] = 0.045–0.071), standardized root mean square residual (SRMR) = 0.045.

**Conclusion:**

By exploring these variables, this study seeks to contribute to the design of more inclusive and sensitive strategies that address the comprehensive and respectful needs of the elderly, thereby improving their quality of life.

## Graphical abstract

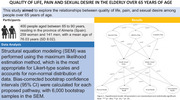

## 1 Introduction

It is unquestionable that in recent decades, there has been a worldwide increase in the population aged 65 years and older (Instituto Nacional de Estadística (INE) 2024; Marzo et al. 2023). Social policies have thus focused on improving the quality of life for this demographic (Marzo et al. 2023).

The World Health Organization (WHO) provides the most widely accepted definition of quality of life, describing it as “the individual's perception of their position in life, in the context of the culture and value systems in which they live and in relation to their goals, expectations, standards and concerns.” This definition also includes the experience of healthy sexuality (The Whoqol Group 1998; de Souza‐Júnior et al. 2021).

Sexuality is a multifaceted and dynamic construct that encompasses behaviors, functions, motivation, partnerships, attitudes, sexual activity, intimacy, pleasure, eroticism, reproduction, beliefs, values, practices, fantasies, identity, desires, roles, and relationships (de Souza‐Júnior et al. 2021; Srinivasan et al. 2019; Lindau et al. 2007; Heath 2019; Lindau et al. 2003). It transcends purely physical and sexual aspects, embracing various qualitative dimensions that contribute to individual well‐being.

Sexuality is an innate aspect of human existence that accompanies individuals from conception to birth and throughout the life cycle until death (Dominguez and Barbagallo 2016; Pérez Martínez 2008; Polonio‐López 2007). Although its expression may change over time, its significance remains constant across all stages of life. In elderly, sexuality continues to play a crucial role in well‐being, offering opportunities for empowerment, affirmation, happiness, and fulfillment (de Souza‐Júnior et al. 2021; Heath 2019). Research has shown that older adults continue to be sexually active; however, various factors, including physical and mental health, gender, partner availability, previous levels of sexual activity, and sociocultural attitudes, can influence how sexuality is experienced in later life. Recognizing and addressing these factors are essential for promoting a holistic understanding of sexual health as an integral component of aging and overall quality of life (Srinivasan et al. 2019; Wang et al. 2015; Lindau and Gavrilova 2010).

Despite its broad significance, much of the existing research on sexuality in older adults has traditionally been limited to the physiological impacts of aging and the medical model of sexuality, often focusing on sexual response and dysfunction (de Souza‐Júnior et al. 2021; Srinivasan et al. 2019). However, a growing body of literature has shifted toward a more holistic, biopsychosocial, and interpersonal approach to sexual well‐being (Srinivasan et al. 2019; Sinković and Towler 2019; Byers and US 2014). Studies increasingly highlight the positive impact of sexual activity and intimacy on interpersonal relationships, physical and mental health, and overall quality of life (Srinivasan et al. 2019; Lindau and Gavrilova 2010; Bell et al. 2017). Recognizing and addressing barriers to healthy sexual expression can further enhance well‐being among older adults and their partners, underscoring the need for a more comprehensive perspective on sexuality throughout the lifespan (Srinivasan et al. 2019).

One of the factors that can influence quality of life is an individual's sexual activity. Research indicates that sexual activity positively influences health (Curley and Johnson 2022; Ravanipour et al. 2013; von Humboldt et al. 2024); however, it also becomes a medical and social challenge as it is often associated with aging (Clayton and Harsh 2016; Kaya et al. 2017; Yee 2010).

Sexual desire, a biological component of sexuality, reflects how individuals express thoughts, attitudes, activities, desires, relationships, and fantasies, influenced by biological, socio‐economic, psychological, cultural, religious, spiritual, ethical, and communicative factors (Gorguet 2008). Sexual desire is defined as the impulse, interest, or motivation to engage in sexual activity, either individually or with another person. It is a natural and normal part of human life, varying significantly across individuals and influenced by factors such as physical health, hormonal changes, psychological states, sociocultural norms, personal relationships, past experiences, and even socioeconomic status (Ravanipour et al. 2013; Faus‐Bertomeu and Gómez‐Redondo 2017; Gómez‐Zapiain 2008; Spector et al. 1996).

Spector et al. (1996) proposed a theory in which sexual desire is divided into two main dimensions: dyadic sexual desire, which refers to the interest in engaging in sexual activities with another person, such as a partner or attractive individual (Gómez‐Zapiain 2008; Spector et al. 1996; Peixoto 2023); and solitary sexual desire, which pertains to the interest in engaging in sexual activities alone, often associated with sexual exploration and independence (Spector et al. 1996; Peixoto 2023).

Chronic medical conditions, fatigue, pain, and decreased mobility have been identified as factors that may influence sexual desire in institutionalized individuals over the age of 65 (Chávez‐Martínez et al. 2018; Lucas‐Matheu and Cabello‐Santamaría 2009; del R 2013). Depression, stress, and anxiety are additional factors that can reduce interest in sex for people of any age, including those over 65. Moreover, certain medications, such as antidepressants and antihypertensives, have side effects that may diminish sexual desire (Vercellino and Philippi 2020).

In women, menopause results in hormonal changes that impact sexual desire (García‐Cruz and Alcaraz 2020; Scavello et al. 2019). In men, age‐related reductions in testosterone levels can affect both sexual desire and erectile function. Furthermore, institutionalized elderly people may experience the loss of partners or close friends, which can reduce their desire and willingness to engage in sexual activities (García‐Cruz and Alcaraz 2020).

Social and cultural attitudes toward sexuality in older age may also play a role in shaping how older people perceive and express their sexual desires (Torres‐Mencía and Rodríguez‐Martín 2019). As individuals age, their willingness to discuss sexual desire often decreases (Ravanipour et al. 2013). Some publications suggest that sexual desire is maintained as people age, not impeding sexual performance, thus highlighting the importance of educating the population about the physical and psychological changes that accompany aging (Alfaro 2014). However, other studies indicate that sexual desire decreases with age (de Andrade‐Palmeira 2021).

Historically, sexual health in older people has been a taboo subject or considered a non‐essential lifestyle concern outside the scope of the medical field. However, increased life expectancy, the recognition of sexual health as an integral part of general well‐being, and the availability of medications to improve sexual function have led to a shift in attitudes. Both older people and the medical community are now more willing to address these concerns, resulting in an increased demand for assistance in this area (Villafuerte‐Reinante et al. 2017).

Sexual dysfunction is highly prevalent in older people. In women, the most common issues include pain disorders, arousal disorders, orgasmic disorders, and hypoactive sexual desire disorder. In men, premature ejaculation, delayed ejaculation, and erectile dysfunction are among the most frequently reported conditions (Dominguez and Barbagallo 2016; Scavello et al. 2019; Llover and Jiménez 2017).

Individuals over the age of 65, particularly those in institutional care, often face additional barriers to expressing their sexuality. These challenges include physical and mental health issues, lack of privacy, and cultural and social attitudes. It is essential for healthcare professionals and institutional staff to receive training on addressing sexual desire in institutionalized patients with understanding, sensitivity, and respect, while recognizing their dignity and autonomy (Romero‐de San Pío et al. 2021).

Among the different factors affecting sexuality in older adults, pain is particularly significant due to its high prevalence and its impact on daily functioning, which can directly influence sexual desire in individuals over the age of 65. Pain is recognized as an important public health issue, ranking among the leading reasons for healthcare consultations and exerting a notable economic impact on the healthcare sector (Guillén and Zúñiga 2022). The experience of pain is unique and subjective, varying by individual and shaped by physical, psychological, biological, social, and cultural factors (Guillén and Zúñiga 2022; Cuenda‐Gago and Espejo‐Antúnez 2017).

Chronic pain and its treatments can negatively affect both sexual functioning and satisfaction. Ruiz‐Palomino et al. (2022) emphasized the importance of assessing and addressing sexuality when treating individuals with chronic pain. Several studies highlight the critical role of sexual desire and the detrimental impact its loss can have on quality of life, emphasizing the need to inquire about sexual desire during consultations in order to identify issues and determine appropriate best treatment (Holloway and Wylie 2015; Birke et al. 2019).

Although research on sexuality in older adults has expanded in recent years, the literature has extensively explored the relationship between chronic pain and sexual dysfunction (Dominguez and Barbagallo 2016; Clayton and Harsh 2016; Ruiz‐Palomino et al. 2022; Avis 2000; Drummond et al. 2013), whereas few studies have examined its specific impact on sexual desire and its broader implications for quality of life (Holloway and Wylie 2015; Birke et al. 2019; Van Overmeire et al. 2021). Considering that desire is a central component of sexual experience and that chronic pain is highly prevalent among older adults, its potential to negatively impact both physical and psychological well‐being underscores the need for a deeper understanding of this relationship. Gaining insight into this interaction is essential for developing more effective healthcare strategies and interventions aimed at improving overall quality of life in this population.

Research on changes in sexual desire among older people presents mixed findings (Curley and Johnson 2022; Ravanipour et al. 2013; von Humboldt et al. 2024; de Andrade‐Palmeira 2021; Holloway and Wylie 2015; Flynn and Gow 2015; Schaller et al. 2023). Although an increasing number of studies examine sexuality in old age, few provide an in‐depth exploration of sexual desire at this stage, with many focusing on it from a pathological perspective (García‐Cruz and Alcaraz 2020; Birke et al. 2019; Van Overmeire et al. 2021; Monteagudo et al. 2016). This study addresses this gap, aiming to explore the relationship among quality of life, pain, and sexual desire in people over 65 years of age. Unlike the previous research, which has often approached sexuality in older adults from a pathological perspective, this study takes a broader approach, considering the psychological and social dimensions of sexual well‐being.

We hypothesize that there is a relationship among sexual desire, pain levels, and health‐related quality of life in people over the age of 65.

- Negative relationship between pain levels and sexual desire.
- Positive relationship between sexual desire and health‐related quality of life.
- Negative relationship between pain levels and health‐related quality of life.

## 2 Methods

### 2.1 Design

This study employed a cross‐sectional design to examine institutionalized and non‐institutionalized people over 65 years of age.

### 2.2 Participants

A convenience sampling method was used, consisting of 400 people aged between 65 and 90 years residing in the province of Almería (Spain). Half of them were institutionalized. The province of Almería was chosen for this study due to its demographic characteristics, which make it a relevant setting for exploring the relationship among quality of life, pain, and sexual desire in older adults. According to data from the National Institute of Statistics, 17.9% of Almería's population is over 65 years of age, and this proportion has been steadily increasing over the past decade (Instituto de Estadística y Cartografía de Andalucía 2024; Instituto Nacional de Estadistica 2024 ). The study's findings could be relevant for understanding aging in community‐dwelling and institutionalized older adults within this specific socio‐demographic context (Zunzunegui 2022).

The sample included 259 women and 141 men, with a mean age of 76.03 years (standard deviation [SD] 8.02). Participants were required to meet the following inclusion criteria:

• Aged 65 years or older.
• Free from cognitive impairment.
• Able to read and write.
• Physically and mentally capable of completing the questionnaires.
• Willing to provide informed consent.
• Either institutionalized in a residential center or residing in Almería or its province.

Exclusion criteria were refusal to sign the informed consent to participate in the research study and obtaining a score of less than 6 on the clock test.

According to the last CENIE report of 2024, the number of people aged 65+ in Almería was 203,741. On the basis of the precepts established by Krejcie and Morgan (1970) where taking into account the maximum admitted margin of error of 5%, the minimum representative sample would be 238 participants, this number representing a confidence level interval of 95%.

### 2.3 Instruments

The *Inventario del Deseo Sexual* (Sexual Desire Inventory) is a 13‐item self‐administered questionnaire validated in Spanish by Ortega et al. (2006). It is based on the original *Sexual Desire Inventory* (Spector et al. 1996). Items are rated on a Likert scale, ranging from 0 (no desire) to 8 (strong desire), with certain items scored based on frequency and importance. The first nine items assess dyadic sexual desire, whereas items 10–13 assess solitary sexual desire. The Spanish version has demonstrated reliability coefficients of 0.87 for dyadic sexual desire and 0.88 for solitary sexual desire (Ortega et al. 2006).

The Cuestionario de Dolor Español (CDE) (Ruiz‐López et al. 1991) (Spanish Pain Questionnaire) is a self‐administered questionnaire targeted at individuals experiencing acute or chronic pain. It evaluates pain across three dimensions:

• Sensorial: Temporal, thermal, constrictive pressure, punctate pressure, incisive pressure, gravitative traction pressure, spatial, and vividness.
• Affective: Fear, autonomic, vegetative, punishment, tension, tiredness, anger, disgust, grief, and anxiety.
• Evaluative: Temporal and intensity dimensions.

The Spanish version of the CDE has reliability coefficients of 0.74 for total pain, 0.73 for sensory pain, and 0.56 for affective pain, with no items measuring evaluative pain (Lázaro et al. 1994). Subsequent studies, such as those by Camacho et al. (2002), have reported improved reliability coefficients of 0.84.

The WHOQOL‐BREF quality of life scale (The Whoqol Group 1998) consists of 26 items with five response options. It provides an overall score related to the perception of total quality of life and scores for each domain: physical health, psychological health, social relationships, and environment. Partial sums are obtained for each domain, in scores ranging from 0 to 100, with higher scores indicating better quality of life (The Whoqol Group 1998).

The questionnaire has been validated in Spain (Lucas‐Carrasco 1998) with a sample of 558 participants. The dimension coefficients ranged from 0.69 to 0.90. The scores generated by the WHOQOL‐BREF showed correlations with the WHOQOL‐100, approximately 0.90. Furthermore, the instrument exhibited good discriminant, content, and test–retest validity (de Oliveira‐Neto 2008).

### 2.4 Procedure

In 2017, the different private and public residential centers in the province of Almeria were invited to participate in the study. Invitations were sent via letters, emails, or meetings with center directors. Several meetings were held with staff to recruit participants.

Additional participants were recruited from centers for active participation and private homes of elderly people. The questionnaires were self‐administered, and assistance was provided upon request.

The sample included both institutionalized and non‐institutionalized individuals, as the Sexual Desire Inventory evaluates not only dyadic sexual desire but also solitary sexual desire. Although institutionalized individuals may have fewer opportunities for partnered relationships, they still experience sexual desire. Given that the study aimed to assess sexual desire in people over 65 years old, it was essential to ensure that institutionalized individuals—who represent a significant percentage of this age group—were included.

### 2.5 Data Analysis

First, descriptive statistical analyses were conducted, including means, SDs, kurtosis, and Pearson correlations. Subsequently, reliability was assessed using three parameters: (a) Reliability analysis was performed using the *ω* coefficient and Cronbach's *α*. These indices examine item measurement errors and their association with their constructs (Trigueros and García‐Mas 2025); (b) the average variance extracted (AVE) (Fornell and Larcker 1981) was calculated. This index distinguishes between factor variance free of measurement error (true variance) and variance affected by measurement errors (error variance). Convergent validity of the factor is not considered valid when values are below 0.50. (III) Finally, the heterotrait–monotrait (HTMT) ratio of latent factor correlations was computed, assuming its discriminant capability when values are below 0.90. This method has been shown to be effective in terms of specificity and sensitivity compared to other techniques, especially in large samples (Henseler et al. 2016).

Second, a structural equation modeling (SEM) approach was used to analyze the relationships established among the study variables. The maximum likelihood estimation method was employed for SEM, as it is the most appropriate for Likert‐type scales and accounts for the non‐normal distribution of the data. Bias‐corrected bootstrap confidence intervals (95% CIs) were calculated for each proposed path, using 6000 bootstrap samples in the SEM (Hayes and Scharkow 2013). Model fit was evaluated using the fit indices and criteria established by Hair et al. (2010). Specifically, SRMR and RMSEA were considered acceptable when values were equal to or lower than 0.06. Incremental indices, including Tucker Lewis Index (TLI), Incremental Fit Index (IFI), and Comparative Fit Index (CFI), were deemed acceptable when values exceeded 0.95. The chi‐square/degrees of freedom ratio (*χ* ^2^/df) indicated a good fit when values ranged between 2 and 3. In addition, effect sizes were included through analysis of variance (*R* ^2^).

**FIGURE 1:**
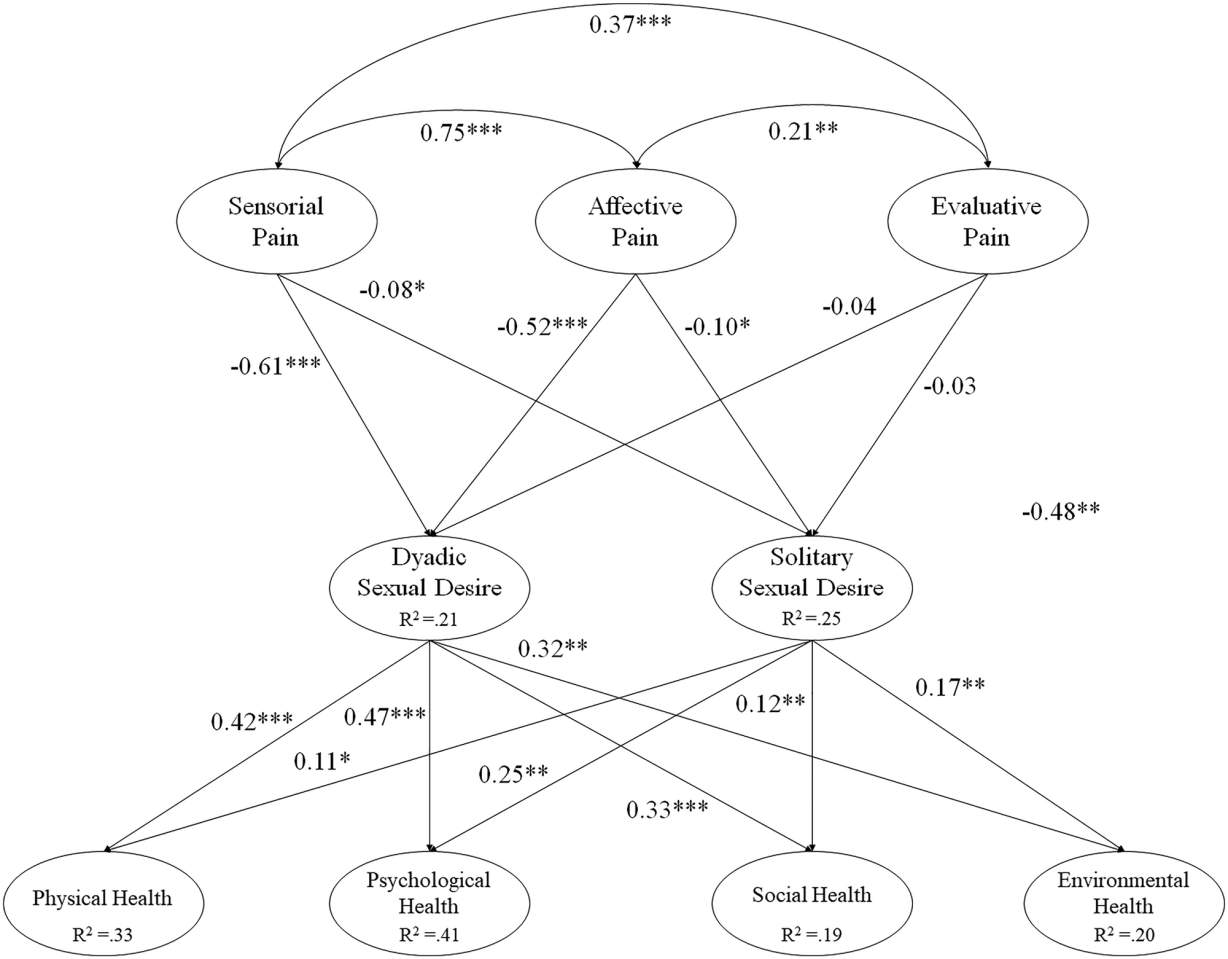
Hypothesized model. All parameters are standardized and statistically significant. ***p < 0.001; **p < 0.01; *p < 0.05.

Despite highlighting the need to analyze the relationships between the variables in the hypothesized model (see Figure 1), it was important to examine two alternative models. In the first alternative model, the three factors related to pain were combined into a single factor. In the second model, the four health‐related factors were grouped into one. Two values will be used to compare the models: (a) the Akaike information criterion (AIC), which estimates the prediction error and, therefore, the relative quality of statistical models for a given dataset; (b) the Bayesian information criterion (BIC), which serves as a model selection criterion within a finite set.

## 3 Results

### 3.1 Preliminary Analysis

Table 1 presents the scores related to descriptive statistics (means, standard deviations, and bivariate correlations), kurtosis values, reliability analyses (Cronbach's *α* and *ω* index), and discriminant validity through HTMT scores.

**TABLE 1:** Descriptive statistics, bivariate correlations, reliability analysis, and HTMT.

Factors	*M*	SD	*k*	*α*	*ω*	AVE	1	2	3	4	5	6	7	8	9
1. Sensory pain	2.96	2.57	1.01	0.86	0.84	0.49	—	0.85^***^	0.37^***^	−0.18^**^	−0.08	−0.45^***^	−0.25^***^	−0.11^*^	−0.05
2. Affective pain	2.32	2.18	1.12	0.83	0.82	0.53	0.72	—	0.28^**^	−0.14^*^	−0.08	−0.44^***^	−0.27^***^	−0.12^*^	−0.08
3. Evaluative pain	0.82	0.38	0.95	0.80	0.80	0.52	0.29	0.25	—	−0.04	−0.03	−0.05	−0.00	0.07	0.09^*^
4. Dyadic desire	40.25	16.74	0.84	0.81	0.78	0.55	0.16	0.12	0.10	—	0.53^**^	0.30^***^	0.44^***^	0.36^***^	0.20^***^
5. Solitary desire	10.66	10.67	1.26	0.80	0.82	0.54	0.28	0.11	0.11	0.48	—	0.15^***^	0.23^***^	0.14^***^	0.15^**^
6. Physical health	13.32	3.09	1.47	0.79	0.80	0.51	0.10	0.15	0.12	0.28	0.11	—	0.66^***^	0.53^**^	0.54^***^
7. Psychological health	13.28	2.70	0.98	0.83	0.83	0.56	0.24	0.31	0.33	0.40	0.20	0.61	—	0.62^***^	0.64^***^
8. Social health	13.03	3.30	1.33	0.84	0.84	0.53	0.16	0.20	0.21	0.31	0.21	0.46	0.58	—	0.57^***^
9. Environmental health	13.58	2.40	1.00	0.82	0.81	0.59	0.14	0.24	0.25	0.36	0.42	0.50	0.60	0.52	—

*Note*: The values below the diagonal correspond to the heterotrait to monotrait (HTMT) ratio between factors.

Abbreviations: AVE, average variance extracted; HTMT, heterotrait–monotrait; *k*, kurtosis values; *M*, means; SD, standard deviation; *α*, Cronbach's *α* index; *ω*, Cronbach's *ω* index.

*** *p *< 0.001.

** *p *< 0.01.

* *p *< 0.05.

Reliability analyses (Cronbach's *α* and *ω* index) showed scores above 0.70, suggesting good internal consistency (Brown 2002). Statistically significant positive correlations were observed between sexual desire and quality of life factors, indicating that higher quality of life is associated with higher sexual desire and vice versa.

Sexual desire demonstrated statistically significant negative correlations with certain pain factors, indicating that higher sexual desire is associated with lower levels of pain. Additionally, negative correlations were found between quality of life and specific factors, particularly in the domains of physical health and pain.

### 3.2 Structural Equation Model

Table 2 shows the fit indices for both the hypothesized model and the two alternative models. The fit indices were good for both the hypothesized model and the first alternative model. However, the second alternative model did not show acceptable fit indices.

**TABLE 2:** Fit indices of the three structural equation models.

	*χ* (Marzo et al. 2023)	df	*χ* (Marzo et al. 2023)/df	RMSEA (90% CI)	SRMR	CFI	IFI	TLI	AIC	BIC
Hypothesized model	3117.61	1259	2.48	0.058 (CI = 0.045–0.071)	0.045	0.96	0.96	0.96	19561.33	19880.78
First alternative model	2330.46	726	3.21	0.064 (CI = 0.059–0.070)	0.051	0.95	0.95	0.95	18879.32	18891.03
Second alternative model	936.51	162	5.78	0.089 (CI = 0.084–0.0.95)	0.061	0.90	0.90	0.90	8369.14	8372.03

Abbreviations: AIC, Akaike information criterion; BIC, Bayesian information criterion; CFI, Comparative Fit Index; CI, confidence interval; IFI, Incremental Fit Index; RMSEA, root mean square error of approximation; SRMR, standardized root mean square residual; TLI, Tucker Lewis Index.

To ensure the adequacy of the hypothesized model, two alternative models were tested. In the first alternative model, the three pain‐related factors (sensory, affective, and evaluative pain) were combined into a higher order factor called pain. This first alternative model showed acceptable fit indices. However, when compared to the hypothesized model, the *χ* ^2^/df and RMSEA fit statistics were lower in the hypothesized model, and there were higher values for the incremental indices compared to alternative model one. As for the second alternative model, the four health‐related factors (physical, psychological, social, and environmental health) were combined into one higher order factor called General Health. This second alternative model did not show acceptable fit indices. Thus, the hypothesized model showed lower values of *χ* ^2^/df and RMSEA, and higher values of CFI, IFI, and TLI compared to this second alternative model. These analyses reinforce the relevance of the proposed model, which allows us to analyze the relationship between the variables under study.

The relationships among the different factors included in the hypothesized model are described below (see Figure 1).

- Sensory pain was positively correlated with affective pain (*β* = 0.75, *p* < 0.001) and evaluative pain (*β* = 0.57, *p *< 0.001). Similarly, affective pain was positively correlated with evaluative pain (*β* = 0.21; *p* < 0.01).
- Sensory pain was negatively associated with dyadic sex (*β* = −0.61, *p* < 0.001) and solitary sex (*β* = −0.08, *p* < 0.05). Similarly, affective pain was negatively associated with dyadic sex (*β* = −0.52, *p* < 0.001) and solitary sex (*β* = −0.10, *p* < 0.05). Evaluative pain was negatively associated with dyadic sex (*β* = 0.04, *p* < 0.41) and solitary sex (*β* = −0.03; *p* < 0.39).
- Dyadic sex was positively associated with physical health (*β* = 0.42, *p* < 0.001), psychological health (*β* = 0.47, *p* < 0.001), social health (*β* = 0.33, *p* < 0.001), and environmental health (*β* = 0.32, *p* < 0.001). Solitary sex was positively associated with physical health (*β* = 0.11, *p* < 0.05), psychological health (*β* = 0.25, *p* < 0.01), social health (*β* = 0.12, *p* < 0.01), and environmental health (*β* = 0.17, *p* < 0.01).

## 4 Discussion

Sexuality influences human health across all stages of life, affecting fundamental biological, psychological, social, and cultural aspects (Dominguez and Barbagallo 2016). The hypothesized model in this study describes that higher levels of pain (sensory, affective, and evaluative) are associated with lower sexual desire (both dyadic and solitary). Pain may reduce sexual motivation due to physical discomfort, psychological distress, or medication side effects. Higher sexual desire (both dyadic and solitary) is associated with better health‐related quality of life, including physical, psychological, social, and environmental well‐being. Engaging in sexual thoughts or activities may contribute to emotional satisfaction, social connection, and overall well‐being.

The literature confirms that sexuality is closely linked to quality of life (de Souza‐Júnior et al. 2021; Rodrigues et al. 2018; Vieira et al. 2016), playing a significant role over the years by contributing to self‐awareness, well‐being, pleasure, and self‐esteem (de Souza‐Júnior et al. 2021; von Humboldt et al. 2020). It also supports mental health maintenance and overall life satisfaction (de Souza‐Júnior et al. 2021; Jackson et al. 2019).

It is important to recognize that sexuality in older people is a natural and significant aspect of life and should be integrated into gerontological studies (Fredriksen‐Goldsen 2023). Sexuality persists in old age but manifests differently for each individual, shaped by personal circumstances. Quality of life and overall life satisfaction are influenced by the emotional and sexual experiences of older adults, who regard sexuality as a significant aspect of their lives (de Souza‐Júnior et al. 2021; Srinivasan et al. 2019). Addressing sexual desire in individuals over the age of 65 requires openness, respect, and understanding (Dominguez and Barbagallo 2016; von Humboldt et al. 2024).

Although the frequency of sexual intercourse tends to decrease among older adults (de Souza‐Júnior et al. 2021; Lindau et al. 2007), this does not signify the end of sexual expression. Therefore, it is essential to recognize that aging does not equate to asexuality but rather represents a stage of life in which sexuality adapts to the unique characteristics of this period (de Souza‐Júnior et al. 2021).

Although it is common in developed countries to reach old age, not all people are able to do so with an optimal quality of life. Factors such as mental health and functional capacity significantly influence how older people perceive their health status and quality of life (Azpiazu‐Garrido et al. 2002; Silva et al. 2017).

The present study found a positive correlation between quality of life and sexual desire—both dyadic and solitary—indicating that better quality of life is associated with higher sexual desire and vice versa. This finding aligns with the previous research that highlights the positive impact of social relationships and mood on increasing sexual desire (Carrasco et al. 2018). Specifically, the social domain of quality of life appears to play an important role in enhancing sexual desire. Moreover, regular engagement in sexual activities contributes not only to greater relationship satisfaction but also to overall physical and psychological well‐being. Studies have shown that sexual activity is associated with lower rates of depressive symptoms, improved self‐esteem, and better cardiovascular health in both men and women (de Souza‐Júnior et al. 2021; DeLamater and Koepsel 2015). Additionally, sexual expression reinforces personal identity, affirms individual values, and fosters feelings of affection, warmth, and love, all of which further enhance well‐being and life satisfaction in older adults (de Souza‐Júnior et al. 2021).

The bidirectional nature of this relationship is also worth considering. However, higher quality of life may foster greater sexual interest and activity, and engaging in sexual expression can, in turn, enhance quality of life by reinforcing emotional connections, boosting self‐esteem, and providing physical and psychological pleasure (DeLamater and Koepsel 2015; DeLamater 2012). This aligns with studies showing that older adults who maintain a satisfying sex life report better mood, reduced stress levels, and lower rates of depression and anxiety (DeLamater and Koepsel 2015). These findings underscore the need to integrate sexual health into gerontological care, ensuring that sexuality is recognized as a fundamental aspect of aging rather than a marginal or taboo topic.

Our results also demonstrate a negative correlation between sexual desire and pain, meaning that higher levels of pain are associated with lower sexual desire and vice versa. The interplay between pain and sexuality significantly influences the quality of life in older adults, affecting both their sexual desire and overall well‐being (Ruiz‐Palomino et al. 2022; Romera‐Baures 2018). Pain is a common issue among the elderly, often resulting from chronic conditions such as arthritis, neuropathy, or other age‐related ailments (Romera‐Baures 2018; Cherpak and dos Santos 2016). This persistent discomfort can lead to significant physical limitations, altering how individuals perceive their bodies and sexual desire. Existing literature underlines the negative impact of pain on the quality of life of the elderly, noting that pain is highly prevalent in this population (Ruiz‐Palomino et al. 2022; Romera‐Baures 2018; Cherpak and dos 2016; de Luna‐Rodríguez et al. 2020). Inadequately treated pain leads to significant economic, personal, social, and familial financial burdens. The elderly experience a higher incidence of painful conditions, highlighting the importance of implementing effective strategies for pain assessment and management. In addition, hospital admissions among people over 65 are three times more frequent than among younger patients (de Luna‐Rodríguez et al. 2020).

In‐line with the previous research, such as Ruiz‐Palomino et al. (2022), the results suggest that among older men, there is a stronger relationship between sexual desire and higher quality of life. This disparity may be explained by a combination of biological, social, and cultural factors. Biologically, age‐related hormonal changes, such as the decline in testosterone levels in men and estrogen in women, may affect sexual desire differently (Ravanipour et al. 2013; García‐Cruz and Alcaraz 2020; Monteagudo et al. 2016). Socially, gender norms and expectations regarding sexuality in older adulthood may influence the way men and women perceive and report their sexual desire. This same study also highlighted that health problems in people over the age of 55 significantly affect their quality of life (Ruiz‐Palomino et al. 2022), a finding that is consistent with the data obtained in the present study. Specifically, negative correlations were observed between quality of life—particularly in the domain of physical health—and pain, corroborating that higher levels of pain are associated with lower quality of life.

The results show that physical health positively influences quality of life and sexual desire, validating the initial hypothesis of this study. This finding highlights the importance of promoting holistic health in older people not only to enhance their overall well‐being but also to maintain their vitality and sexual desires as integral aspects of healthy aging.

Among the limitations of this study, it should be noted that, although the sample size was considerable, future research would benefit from expanding the sample to include participants from different geographical regions and conducting a multicenter study across different provinces in Spain. Furthermore, most of the participants identified as heterosexual, and a significant proportion of the women came from middle‐ to upper class socioeconomic backgrounds, with university education or professional careers. These characteristics may limit the generalizability of the findings. Additionally, the study relies on self‐reported data, which may be subject to bias, particularly in sensitive topics such as sexuality. Moreover, the role of social and emotional well‐being in sexual desire should be further explored.

Although our findings highlight a negative correlation between pain and sexual desire, the underlying mechanisms of this relationship warrant further exploration. Chronic pain not only causes physical discomfort but is also associated with psychological distress, including anxiety and depression, which can further diminish sexual motivation. Additionally, the use of pain management medications, such as opioids and certain antidepressants, may have adverse effects on sexual function, contributing to reduced desire (Birke et al. 2019). Mobility limitations resulting from pain could also play a role by restricting physical closeness and intimacy, which are essential components of sexual relationships.

Another limitation of this study is that information regarding partner availability or the recency of the last sexual encounter was not collected, as the validated questionnaire used did not include specific items addressing this aspect. Given the potential influence of these factors on dyadic sexual desire, future research would benefit from incorporating questions related to partner availability and recent sexual activity. This would allow for a more comprehensive understanding of the contextual factors that may shape sexual desire in older adults.

Future studies should also examine additional variables such as age, gender, level of education, and institutionalization, as these factors could provide valuable insights and inform new lines of research related to sexuality in older people. Such work would contribute to further understanding and improving quality of life for this population. In addition, future research should consider various multidimensional influences to better understand the complex interactions among pain, quality of life, and sexual desire in aging populations. These factors include biological aspects, such as hormonal changes and chronic health conditions; psychological elements, including mental health status and emotional well‐being; social dynamics, such as relationship quality and cultural attitudes toward aging and sexuality; and environmental conditions, including access to healthcare and living situations. Addressing these influences will provide a more comprehensive understanding of the factors affecting sexual health and well‐being in older adults.

### 4.1 Applicability of the Study for Clinical Practice

This study provides a solid foundation for designing educational programs for healthcare professionals to enhance their understanding of sexuality in individuals aged 65 and older. These programs might focus on addressing misconceptions, reducing stigma, and providing strategies for integrating discussions about sexual health into clinical practice. Additionally, they could also be adapted for older adults themselves, promoting awareness and facilitating open conversations about sexual well‐being. The study's findings, particularly the associations among pain, quality of life, and sexual desire, underscore the need for targeted education that considers the multidimensional aspects of sexuality in aging populations.

The study also highlights the potential to expand the sample to include participants under the age of 65, both institutionalized and non‐institutionalized. Additionally, the preliminary findings open avenues for further investigation into the interplay among pain, quality of life, and sexual desire, as well as how these elements are conditioned by overall health status.

In people over 65, sexual desire may be influenced by various physical, emotional, and social factors (Gorguet 2008; Faus‐Bertomeu and Gómez‐Redondo 2017). Limitations that affect sexual desire at this stage of life include hormonal changes, chronic health conditions, medication side effects, and specific issues such as erectile dysfunction in men or vaginal dryness in women (Nuñez Rodriguez 2013; García‐Cruz and Alcaraz 2020; Scavello et al. 2019). In addition, psychological factors such as depression, anxiety, and stress may also negatively impact sexual desire (Vercellino and Philippi 2020), highlighting the need to include these variables and additional assessment tools in future studies.

The decline in sexual desire associated with aging (de Andrade‐Palmeira 2021) underscores the importance of investigating how this phenomenon evolves across life stages, considering personal, social, and economic factors. The lack of sex education tailored to older people may contribute to misinformation and hinder the effective management of sexual issues associated with aging (Alfaro 2014). This suggests a need to develop educational programs for health professionals to address these issues comprehensively.

Age‐specific sexuality education for older people could play a crucial role in dispelling myths and reducing stigma surrounding sexuality in later life, while also providing strategies for maintaining a fulfilling sex life during this stage. Integrating health education, including sexual education, into residential facilities or institutions frequented by people over 65 may significantly enhance their well‐being and quality of life. Additionally, primary care programs can serve as an essential resource for identifying risks and promoting optimal sexual health. Finally, consultations with medical specialists who can offer personalized solutions tailored to individual needs are highly recommended.

## 5 Conclusion

In individuals aged 65 and older, sexual desire decreases in the presence of pain, whereas higher quality of life is associated with increased sexual desire. Health status positively influences sexual desire, showing that better health correlates with greater sexual desire in this population.

Statistical analyses reveal a close relationship among sexual desire, quality of life, and pain in people over 65. Specifically, higher levels of pain are associated with reductions in both quality of life and sexual desire.

In conclusion, these findings highlight the complex interplay among sexual desire, quality of life, and pain in people over 65, underlining the need to address these factors in an integrated manner within the geriatric care settings. Implementing strategies focused on promoting overall health and effectively managing pain could substantially improve quality of life and support healthy sexuality, which remains a fundamental component of well‐being in later life.

## Author Contributions

**Patricia Rocamora‐Pérez**: conceptualization, investigation, writing – original draft, writing – review and editing, methodology. **Azahara de la Poza‐Rodríguez**: conceptualization, investigation, methodology, writing – review and editing, writing – original draft, formal analysis. **María Jesús Benzo‐Iglesias**: conceptualization, investigation, writing – original draft, writing – review and editing, methodology. **José Manuel Aguilar‐Parra**: conceptualization, investigation, writing – original draft, methodology, formal analysis, writing – review and editing. **Rubén Trigueros‐Ramos**: conceptualization, investigation, writing – original draft, methodology, writing – review and editing, formal analysis. **Remedios López‐Liria**: conceptualization, investigation, writing – original draft, methodology, writing – review and editing. **María José Morales‐Gázquez**: conceptualization, investigation, writing – original draft, writing – review and editing, methodology.

## Ethics Statement

The study was approved by the Human Research Bioethics Committee of the University of Almeria (reference: UALBIO2017/004).

## Consent

The written informed consent was obtained from all subjects; it was documented and witnessed. The volunteers completed a health questionnaire/report to confirm that they met the criteria proposed. All research procedures were performed in accordance with the 1975 Declaration of Helsinki, and the consent form the questionnaire applied.

## Conflicts of Interest

The authors declare no conflicts of interest.

## Peer Review

The peer review history for this article is available at https://publons.com/publon/10.1002/brb3.70655

## Data Availability

The datasets used and/or analyzed during the current study are available from the corresponding author on reasonable request.
